# The prognosis after contraindicated surgery of NSCLC patients with malignant pleural effusion (M1a) may be better than expected

**DOI:** 10.18632/oncotarget.8566

**Published:** 2016-04-04

**Authors:** Yijiu Ren, Chenyang Dai, Jianfei Shen, Yang Liu, Dong Xie, Hui Zheng, Jiaxi He, Wenhua Liang, Gening Jiang, Ke Fei, Ping Yang, Jianxing He, Chang Chen

**Affiliations:** ^1^ Department of Thoracic Surgery, Shanghai Pulmonary Hospital, Tongji University School of Medicine, Shanghai, People's Republic of China; ^2^ Department of Thoracic Surgery, First Affiliated Hospital of Guangzhou Medical University & Guangzhou Research Institute of Respiratory Disease, Guangzhou, People's Republic of China; ^3^ Division of Epidemiology, Department of Health Sciences Research, Mayo Clinic, Rochester, MN, USA

**Keywords:** lung cancer, surgery, malignant pleural effusion, prognosis, surveillance epidemiology and end-results database

## Abstract

Although non-small cell lung cancer (NSCLC) with malignant pleural effusion (M1a) is generally contraindicated for surgery, several reports have demonstrated favorable prognosis. This study aimed to describe the results of surgical intervention in this disease. In this retrospective study, we evaluated NSCLC patients with ipsilateral malignant pleural effusion selected from Surveillance Epidemiology and End-Results database (SEER). Primary tumor resection was compared to no tumor resection in the overall survival (OS) and lung cancer-specific survival (LCSS). Multivariate analyses and propensity score matching were applied to compare the two groups. The study included 2,217 eligible patients. Primary tumor resection group was significantly associated with better OS and LCSS compared to no tumor resection group (the median survival time (MST), 20 vs 7 months; OS, *p* <0.001; LCSS, *p* <0.001). Multivariable analyses indicated that no primary tumor resection was associated with decreased OS (Hazard Ratio (HR), 2.136; p<0.001) and LCSS (HR, 2.053; p<0.001). In propensity score-matched pairs, better OS and LCSS were further validated in patients with ipsilateral malignant pleural effusion who underwent primary tumor resection compared to no tumor resection (MST, 20 vs 6 months; OS, *p* <0.001; LCSS, *p* <0.001). Similarly, multivariable analyses also indicated that no primary tumor resection was associated with decreased OS (HR, 2.309; *p* <0.001) and LCSS (HR, 2.301; *p* <0.001) for patients with ipsilateral malignant pleural effusion. In conclusion, the prognosis after contraindicated surgery of NSCLC patients with malignant pleural effusion (M1a) may be better than expected. Thus, subsequent studies should aim to identify patients who could benefit from surgery.

## INTRODUCTION

Malignant pleural effusion, as one kind of non-small-cell lung cancer (NSCLC) with pleural dissemination, has been proved to have poor outcomes, and it is generally contraindicated for operations [1–3]. The International Association for the Study of Lung Cancer (IASLC) Staging Project had stated that the median survival time (MST) and the 5-year survival rate of patients with pleural dissemination were 8 months and 2%, respectively [3]. Therefore, NSCLC with malignant pleural effusion was staged as IV (M1a) in the new staging system of the Union for International Cancer Control [4].

Lim and colleagues reported that positive pleural lavage cytology during surgical resection is an independent predictor factor in predicting worse survival of NSCLC patients with a resectable-stage tumor [5]. Ryu and colleagues founded that prognostic impact effect of minimal pleural effusion was higher in early rather than advanced stages of NSCLC [6]. However, recently, many surgeons have reported that the postoperative prognosis of patients diagnosed with malignant pleural disease at thoracotomy is relatively favorable [7–12].

Hence, in the present study, we used the Surveillance, Epidemiology, and End Results (SEER) database to identify the large cohort of NSCLC patients with ipsilateral malignant pleural effusion reported up to date, and evaluated the prognostic correlates of overall survival (OS) and lung cancer-specific survival (LCSS) in this population.

## RESULTS

This study included 2,217 patients with ipsilateral malignant pleural effusion from SEER registry patients diagnosed with NSCLC between 2004 and 2012 with no prior history of malignancy. Of these patients, 128 had primary tumor resection and 2,089 did not have primary tumor resection. The distribution of specific patient and tumor characteristics among patients with ipsilateral malignant pleural effusion is shown in Table 1. Surgeons seem more inclined to perform primary tumor resection in younger M1a patients with malignant pleural effusion and tumor size of less than 7cm.

**Table 1:** Baseline characteristics of M1a NSCLC patients only due to ipsilateral malignant pleural effusion

Characteristics	Primary tumor resection	None primary tumor resection	*p* Value
	N=128	N=2089	
Age group			**<0.001**
≤65 y	65 (50.8)	617 (29.5)	
>65 y	63 (49.2)	1472 (70.5)	
Gender			0.375
Male	75 (58.6)	1140 (54.6)	
Female	53 (41.4)	949 (45.4)	
Married	75 (58.6)	1022 (48.9)	0.034
Race/ethnicity			0.569
White	100 (78.1)	1624 (77.7)	
Black	19 (14.8)	268 (12.8)	
Other	9 (7.0)	197 (9.4)	
Tumor Size			**<0.001**
≤3cm	56 (43.8)	548 (26.2)	
≤5cm	35 (27.3)	598 (28.6)	
≤7cm	17 (13.3)	490 (23.5)	
>7cm	20 (15.6)	453 (21.7)	
Location			0.292
Main bronchus	10 (7.8)	150 (7.2)	
Upper	55 (43.0)	1104 (52.8)	
Middle	9 (7.0)	110 (5.3)	
Lower	51 (39.8)	687 (32.9)	
Overlap	3 (2.3)	38 (1.8)	
Lymph node status			**<0.001**
N0	53 (41.4)	641 (30.7)	
N1	15 (11.7)	155 (7.4)	
N2	58 (45.3)	978 (46.8)	
N3	1 (0.8)	222 (10.6)	
NX	1 (0.8)	93 (4.5)	
Histology			0.386
Adenocarcinoma	66 (51.6)	1042 (49.9)	
Squamous cell carcinoma	34 (26.6)	482 (23.1)	
Other	28 (21.9)	565 (27.0)	
Radiotherapy			0.795
No	94 (73.4)	1512 (72.4)	
Yes	34 (26.6)	577 (27.6)	
Follow-up time, months	20 (1-48)	7 (1-60)	**<0.001**

Bold values corresponds to the comparisons with P < 0.001.

**Figure 1:**
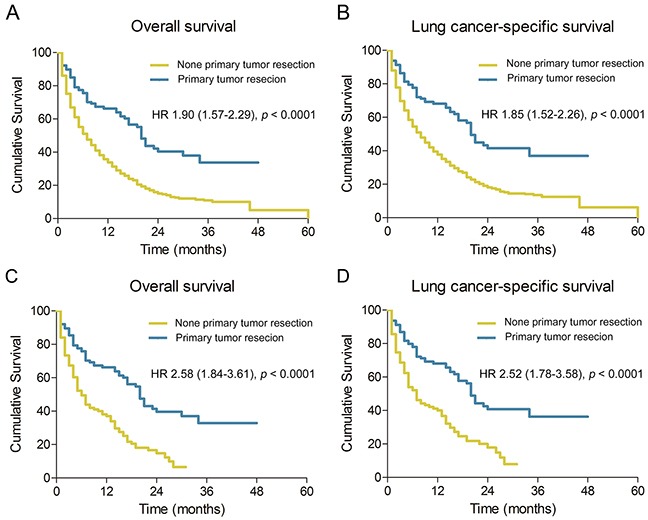
Overall and lung cancer-specific survival in NSCLC patients with ipsilateral malignant pleural effusion before propensity score matching **A.** and **B.**, and NSCLC patients with ipsilateral malignant pleural effusion after propensity score matching **C.** and **D.** HR, hazard ratio; NSCLC, non-small cell lung cancer

Kaplan-Meier analysis showed that for patients with ipsilateral malignant pleural effusion, primary tumor resection group showed significantly better OS and LCSS compared to no tumor resection group (MST, 20 vs 7 months; OS, *p* <0.001; LCSS, *p* <0.001) (Figure 1A, 1B).

Multivariable analyses suggested that among patients with ipsilateral malignant pleural effusion, decreased OS and LCSS is associated with age > 65 years, unmarried, white or black race, and non-radiotherapy (Table 2). Notably, multivariable analyses results indicated that no primary tumor resection was associated with decreased OS (Hazard Ratio (HR), 2.136; 95% CI, 1.645-2.772; *p* <0.001) and LCSS (HR, 2.053; 95% CI, 1.568-2.690; *p* <0.001) for patients with ipsilateral malignant pleural effusion (Table 2).

**Table 2:** Multivariate analysis of overall survival and lung cancer-specific survival in M1a NSCLC patients only due to ipsilateral malignant pleural effusion

Variables	Overall survival		Lung cancer-specific survival	
	Hazard Ratios (95% CI)	*p* Value	Hazard Ratios (95% CI)	*p* Value
Age				
≤65 y	1.00 (Reference)		1.00 (Reference)	
>65 y	1.412 (1.259-1.585)	**<0.001**	1.368 (1.214-1.543)	**<0.001**
Gender				
Female	1.00 (Reference)		1.00 (Reference)	
Male	1.118 (1.008-1.241)	0.035	1.086 (0.974-1.212)	0.138
Married				
Yes	1.00 (Reference)		1.00 (Reference)	
No	1.322 (1.192-1.466)	**<0.001**	1.279 (1.147-1.426)	**<0.001**
Race				
White	1.00 (Reference)		1.00 (Reference)	
Black	0.968 (0.831-1.127)	0.672	0.965 (0.822-1.134)	0.668
Other	0.628 (0.517-0.762)	**<0.001**	0.612 (0.499-0.752)	**<0.001**
Tumor Size				
≤3cm	1.00 (Reference)		1.00 (Reference)	
≤5cm	0.989 (0.862-1.135)	0.880	0.999 (0.865-1.155)	0.994
≤7cm	1.144 (0.986-1.327)	0.075	1.152 (0.985-1.347)	0.077
>7cm	1.339 (1.150-1.560)	**<0.001**	1.348 (1.148-1.583)	**<0.001**
Location				
Main bronchus	1.00 (Reference)		1.00 (Reference)	
Upper	1.021 (0.841-1.241)	0.831	1.041 (0.848-1.279)	0.700
Middle	0.949 (0.717-1.256)	0.713	0.933 (0.693-1.257)	0.649
Lower	0.911 (0.744-1.115)	0.367	0.917 (0.741-1.135)	0.428
Overlap	1.462 (1.012-2.115)	0.043	1.416 (0.955-2.099)	0.084
Lymph node status				
N0	1.00 (Reference)		1.00 (Reference)	
N1	0.880 (0.715-1.082)	0.225	0.871 (0.697-1.088)	0.223
N2	1.156 (1.027-1.301)	0.016	1.216 (1.073-1.379)	0.002
N3	1.076 (0.898-1.291)	0.427	1.130 (0.934-1.367)	0.210
NX	1.134 (0.881-1.459)	0.328	1.226 (0.944-1.591)	0.126
Histology				
Adenocarcinoma	1.00 (Reference)		1.00 (Reference)	
Squamous cell carcinoma	1.258 (1.094-1.447)	0.001	1.255 (1.084-1.453)	0.002
Other	1.360 (1.206-1.533)	**<0.001**	1.317 (1.160-1.495)	**<0.001**
Primary tumor resection				
Yes	1.00 (Reference)		1.00 (Reference)	
No	2.136 (1.645-2.772)	**<0.001**	2.053 (1.568-2.690)	**<0.001**
Radiotherapy				
Yes	1.00 (Reference)		1.00 (Reference)	
No	1.639 (1.447-1.856)	**<0.001**	1.612 (1.415-1.836)	**<0.001**

Bold values corresponds to the comparisons with P < 0.001.

Following the propensity score matching, all variables, including patient characteristics, tumor features, and therapeutic managements, were calculated. Patients with ipsilateral malignant pleural effusion were classified into well matched groups, specifically, 125 patients were assigned to primary tumor resection group and 125 patients to no primary tumor resection group. Table 3 shows no significant difference in demographic, pathologic, and therapeutic variables between two groups. Consistent with the prior analyses, better OS and LCSS were observed among patients with ipsilateral malignant pleural effusion who underwent primary tumor resection when compared to those who did not (MST, 20 vs 6 months; OS, *p* <0.001; LCSS, *p* <0.001) (Figure 1C, 1D). Similarly, multivariable analyses also indicated that no primary tumor resection was associated with decreased OS (HR, 2.309; 95% CI, 1.636-3.259; *p* <0.001) and LCSS (HR, 2.301; 95% CI, 1.610-3.287; *p* <0.001) for patients with ipsilateral malignant pleural effusion (Table 4).

**Table 3:** Baseline characteristics of M1a NSCLC patients only due to ipsilateral malignant pleural effusion after propensity score matching

Characteristics	Primary tumor resection	None primary tumor resection	*p* Value
	N=125	N=125	
Age group			0.800
≤65 y	62 (49.6)	59 (47.2)	
>65 y	63 (50.4)	66 (52.8)	
Gender			0.373
Male	73 (58.4)	60 (48.0)	
Female	52 (41.6)	65 (52.0)	
Married	74 (59.2)	63 (50.4)	0.204
Race/ethnicity			0.939
White	98 (78.4	96 (76.8)	
Black	18 (14.4)	20 (16.0)	
Other	9 (7.0)	9 (9.2)	
Tumor Size			0.908
≤3cm	55 (44.0)	57 (45.6)	
≤5cm	35 (28.0)	32 (25.6)	
≤7cm	15 (12.0)	18 (14.4)	
>7cm	20 (16.0)	18 (14.4)	
Location			0.921
Main bronchus	9 (7.2)	11 (8.8)	
Upper	55 (44.0)	50 (40.0)	
Middle	9 (7.2)	7 (5.6)	
Lower	51 (40.8)	56 (44.8)	
Overlap	1 (0.8)	1 (0.8)	
Lymph node status			0.210
N0	52 (41.6)	53 (42.4)	
N1	14 (11.2)	18 (14.4)	
N2	57 (45.6)	48 (38.4)	
N3	1 (0.8)	6 (4.8)	
NX	1 (0.8)	0 (0)	
Histology			0.662
Adenocarcinoma	65 (52.0)	68 (54.4)	
Squamous cell carcinoma	33 (26.4)	27 (21.6)	
Other	27 (21.6)	30 (24.0)	
Radiotherapy			0.377
No	91 (72.8)	98 (78.4)	
Yes	34 (27.2)	27 (21.6)	
Follow-up time, months	20 (1-48)	6 (1-31)	<0.001

Bold values corresponds to the comparisons with P < 0.001.

**Table 4:** Multivariate analysis of overall survival and lung cancer-specific survival in M1a NSCLC patients only due to ipsilateral malignant pleural effusion after propensity score matching

Variables	Overall survival		Lung cancer-specific survival	
	Hazard Ratios (95% CI)	*p* Value	Hazard Ratios (95% CI)	*p* Value
Age				
≤65 y	1.00 (Reference)		1.00 (Reference)	
>65 y	1.063 (0.746-1.515)	0.735	1.013 (0.700-1.465)	0.947
Gender				
Female	1.00 (Reference)		1.00 (Reference)	
Male	1.233 (0.871-1.745)	0.237	1.226 (0.855-1.758)	0.268
Married				
Yes	1.00 (Reference)		1.00 (Reference)	
No	1.353 (0.946-1.936)	0.098	1.261 (0.870-1.827)	0.220
Race				
White	1.00 (Reference)		1.00 (Reference)	
Black	1.056 (0.643-1.733)	0.831	1.087 (0.652-1.812)	0.748
Other	1.254 (0.632-2.488)	0.517	1.187 (0.583-2.419)	0.636
Tumor Size				
≤3cm	1.00 (Reference)		1.00 (Reference)	
≤5cm	0.670 (0.438-1.024)	0.064	0.622 (0.400-0.968)	0.035
≤7cm	1.157 (0.667-2.007)	0.603	1.048 (0.591-1.856)	0.873
>7cm	0.891 (0.508-1.562)	0.687	0.798 (0.439-1.449)	0.458
Location				
Main bronchus	1.00 (Reference)		1.00 (Reference)	
Upper	0.706 (0.373-1.339)	0.286	0.724 (0.372-1.407)	0.340
Middle	1.012 (0.440-2.328)	0.977	1.012 (0.425-2.419)	0.976
Lower	0.935 (0.485-1.801)	0.840	0.915 (0.461-1.817)	0.801
Overlap	6.215 (1.159-33.325)	0.033	7.041 (1.279-38.772)	0.025
Lymph node status				
N0	1.00 (Reference)		1.00 (Reference)	
N1	1.238 (0.726-2.111)	0.434	1.211 (0.686-2.137)	0.510
N2	1.697 (1.144-2.517)	0.009	1.844 (1.225-2.774)	0.003
N3	2.235 (0.871-5.734)	0.094	2.577 (0.991-6.698)	0.052
NX	<0.001	0.966	<0.001	0.968
Histology				
Adenocarcinoma	1.00 (Reference)		1.00 (Reference)	
Squamous cell carcinoma	0.924 (0.566-1.509)	0.752	1.014 (0.609-1.688)	0.959
Other	1.121 (0.738-1.704)	0.592	1.128 (0.728-1.748)	0.589
Primary tumor resection				
Yes	1.00 (Reference)		1.00 (Reference)	
No	2.309 (1.636-3.259)	**<0.001**	2.301 (1.610-3.287)	**<0.001**
Radiotherapy				
Yes	1.00 (Reference)		1.00 (Reference)	
No	1.275 (0.816-1.992)	0.286	1.391 (0.868-2.230)	0.170

Bold values corresponds to the comparisons with P < 0.001.

## DISCUSSION

In the seventh version of TNM staging for NSCLC, malignant pleural effusion is defined as M1a [13]; therefore, it is generally contraindicated for surgery. However, malignant pleural effusion can be unexpectedly identified during operations when primary tumor removal seems feasible and does not add overloaded pressure to the patients. Dr. Ohta and colleagues reported that for 42 surgically resected cases with confirmed malignant pleural dissemination, 5-year survivals and MST were 13.1% and 17 months, respectively [10]. Dr. Iid and colleagues found that 5-year survival rate and MST of 313 pleural dissemination patients were 29.3% and 34.0 months, respectively. Primary tumor resection was performed in 256 (81.8%) patients, and 152 (48.6%) patients underwent macroscopic complete resection with 5-year survival rates of 33.1% and 37.1%, respectively [12]. In addition, prior results of surgical interventions treating this special disease category provided additional evidence for tumor resection [11, 14]. Therefore, it remains controversial whether attempts should be made to resect the primary tumor when confronting an unexpected malignant pleural effusion case.

Using the SEER database, we analyzed the largest cohort of NSCLC patients with malignant pleural effusion (n = 2,217) reported to date, although the incidence was slightly lower compared to that in previous studies [12, 15]. Also, the analyses of treatment modalities and survival were performed with patients without other M1a and/or M1b disease and with only one primary tumor.

We observed that patients who underwent primary tumor resection, especially NSCLC patients with ipsilateral malignant pleural effusion, collectively exhibited greater OS and LCCS compared to those with no tumor resection (Figure 1). The multivariable analyses showed that primary tumor resection group had a significantly more favorable prognosis compared to no primary tumor resection group (Tables 2, 4). The underlying reason included possible reduction of the tumor burden. Clinical support might be found in a study by Dr. Iid and his colleagues. Considering pleural dissemination patients, they found that 5-year survival rate of macroscopic complete resection group (37.1%) was significantly better compared to macroscopic incomplete resection group (22.7%, *p* = 0.009) and exploratory thoracotomy group (12.2%, *p* < 0.001), respectively [12]. Dr. Ren and colleagues found that for patients with intra-operatively proven malignant nodules and minimal pleural effusion (<300ml), primary tumor resection had better survival rate compared to biopsy (MST, 27 vs 7 months, *p* = 0.003). Theoretical support might be found in Dr. Rashid and his colleagues' study on metastatic breast cancer resection. The authors utilized biotechnology to monitor overall breast cancer load under direct vision mouse model [16] from which they found that only primary tumor resection significantly reduced tumor burden.

Moreover, a relevant published study from Shanghai Pulmonary Hospital also suggested that M1a NSCLC patients with pleural dissemination might not be entirely excluded from the surgery [17]. All surgical NSCLC cases (9,576) of Shanghai Pulmonary Hospital between January 2005 and December 2013 were reviewed. Among them, 83 cases (0.9%) met the definition of “unexpected” macroscopic malignant pleural dissemination, despite routine preoperative evaluations for tumor metastasis. Patients with primary tumor resection had significantly better outcome compared to biopsy (MST: respectively, 35 vs. 17 months, p=0.001). Also, multivariate analysis showed that primary tumor resection (HR: 3.678, p=0.014) were favorable prognostic factors in patients with malignant pleural dissemination.

This study has some limitations. First, the SEER database was generated retrospectively, and inevitably, our analyses of this data are subject to the influence of patient and treatment selection bias. We have attempted to control for this by using some advanced statistical methods to balance the variables between arms. Second, the SEER database set was not integrated for malignant pleural effusion, important factors, such as the pre-operation TNM stage, the amount of pleural effusion, lung function, symptoms, and perhaps the tumor burden. The prognosis for surgery for NSCLC patients with malignant pleural effusion may depend on the factors mentioned above. Because of these limitations, a more complete prospective cohort study is warranted to confirm the role of primary tumor resection in NSCLCs patients, especially in those with intra-operatively proven malignant pleural effusion. Notably, SEER does not provide information about chemotherapy treatment, which is a very fundamental issue in the survival of NSCLC patients with malignant pleural effusion.

Briefly, the results of this study revealed that the prognosis after contraindicated surgery of NSCLC patients with malignant pleural effusion (M1a) may be better than expected. Thus, subsequent studies should aim to identify patients who could benefit from surgery.

## METHODS

### Study population

SEER-18 registry data were used to identify patients that met the inclusion criteria (site = lung and bronchus, behavior = malignant, age = 25-84, and year of diagnosis = 2004-2012) [18]. In addition, we included the patients who had (1) pathologically confirmed NSCLC, (2) M1a disease with ipsilateral malignant pleural effusion (SEER code: CS mets at dx “15”), and (3) only one malignant primary tumor.

We collected the demographic characteristics of patients (age, gender, marriage, and race), pathological features of tumors (size, location, histological type, and lymph node status, location of malignant pleural effusion), and types of therapeutic management (surgical type, and radiotherapy) from SEER database. In this study, pathological types were classified as adenocarcinoma (SEER codes 8140, 8230, 8255, 8260, 8310, 8333, 8470, 8480, 8481, 8490 and 8550), squamous cell carcinoma (SEER codes 8052, 8070, 8071, 8072, 8073, 8083 and 8084), and other histological types with low incidence rate (large cell carcinoma and etc.). Since OS and LCSS were also included in SEER database, both of them were regarded as the outcomes of interest. Patient outcomes were obtained until December 31, 2012. OS was defined as the survival time from diagnosis until death for any reason or until the last follow-up, and LCSS from diagnosis until cause-specific death with lung cancer or until the last follow-up.

### Statistical analysis

The data were presented as frequencies (percent) or median (range) deviation. The comparison of demographic, pathologic, and therapeutic features between patients who underwent primary tumor resection or those who did not was performed using unpaired t test for continuous variables and Pearson χ2 test for categorical variables. The OS and LCSS were estimated using the Kaplan-Meier method and the log-rank test comparing survival in two or more groups. Multivariate Cox proportional hazard analyses were applied to adjust the potential confounders related to patients, tumors, and therapies in the survival analysis. For both Kaplan-Meier and Cox analyses, patients were censored using the SEER.

We used propensity score matching method to balance the differences in the basic clinical characteristics of patients with ipsilateral malignant pleural effusion who underwent primary tumor resection group and those who did not. As an alternative method to compare survival outcomes among surgical procedures, propensity score matching was performed with one-to-three nearest-neighbor matching without replacement to identify matched cohorts representing the two treatment modalities. Specifically, to control the potential difference in the basic clinical characteristics of patients and tumors (variables in the propensity score matching including age, gender, race/ethnical group, lesion site and pathological classification), we made a comparative examination of survival between primary tumor resection group and those who did not to verify better prognosis for patients who underwent primary tumor resection. Covariate balance was evaluated by using standardized differences in means. Finally, OS and LCSS were compared in matched patients with ipsilateral malignant pleural effusion who underwent primary tumor resection group and those who did not by log-rank test.

In this study, a two-sided p value < 0.05 was regarded statistically significant. All analyses were conducted using SPSS 23.0 (SPSS Inc. Chicago, IL), and survival curve was drawn using GraphPad Prism 6.0 (GraphPad Software, San Diego, CA).
